# An atypical case of fatal ‘esophageal apoplexy’: post-mortem findings and differential diagnosis

**DOI:** 10.1007/s00414-024-03280-6

**Published:** 2024-06-27

**Authors:** Hans H. de Boer, Chris O’Donnell

**Affiliations:** Victorian Institute of Forensic Medicine / Department of Forensic Medicine, Monash University, 65 Kavanagh Street, Southbank, VIC 3006 Australia

**Keywords:** Sudden death, Autopsy, Esophagus, Hematoma, Dissection, Esophageal hematoma, Forensic radiology, Forensic pathology

## Abstract

Forensic pathologists need to have comprehensive knowledge of a large variety of causes of sudden natural death. We describe a case of sudden and unexpected death in woman in her sixties due to rupture of a large paraesophageal hematoma. The post-mortem examination and differential diagnosis are discussed. The combined findings of whole-body post-mortem CT imaging (PMCT), targeted PMCT angiography, autopsy, and histology are most in keeping with ‘esophageal apoplexy’; a rare cause of hemorrhage in the esophageal wall. A review of the literature indicates that most cases of esophageal apoplexy are self-limiting and that fatal complications are exceedingly rare. Our case demonstrates that esophageal apoplexy can present as sudden unexpected death.

**Supplementary Information:**

The online version contains supplementary material available at 10.1007/s00414-024-03280-6.

## Introduction

Forensic pathologists are often confronted with cases of sudden unexpected death due to natural causes, and thus require comprehensive knowledge of the presentation and post-mortem findings of a wide variety of potentially fatal natural diseases. We describe a case of sudden and unexpected death of a woman in her sixties due to rupture of a large paraesophageal hematoma. We discuss our post-mortem findings and differential diagnostic process and conclude that the case is best classified as ‘esophageal apoplexy’: a rare and usually self-limiting hemorrhage within the esophageal wall.

## Case circumstances

A female in her mid-sixties with a relevant medical history of gastro-esophageal reflux disease, chronic obstructive pulmonary disease and type 2 diabetes mellitus was found deceased in the hallway of her home. She was last seen alive a day before in apparently in normal health. Scene examination was unremarkable. The death was not deemed suspicious after a police investigation and a reasonable cause of death could not be inferred from the medical history and circumstances. In accordance with local legislation, the case was reported to the Coroners Court of Victoria, Australia. An autopsy was directed to examine the cause of death, which was performed at the Victorian Institute of Forensic Medicine (VIFM).

## Post-mortem radiology

The body was subject to post-mortem CT scan (PMCT). Full body PMCT imaging is performed on all decedents admitted to our Institute using a mortuary-located dual-source Somatom Definition Flash CT scanner (Siemens Healthcare, Erlangen, Germany). The CT imaging technique includes a head-to-toe scan range, at 120 kVp, 280 effective mAs, 1.5 mm slice thickness, pitch of 0.6, rotation time of 0.55 s, 500 mm field of view, and reconstruction kernel of B30f medium smooth. Images were viewed using Syngo.via software (version VB40, Siemens Healthcare, Erlangen, Germany).

**Fig. 1:**
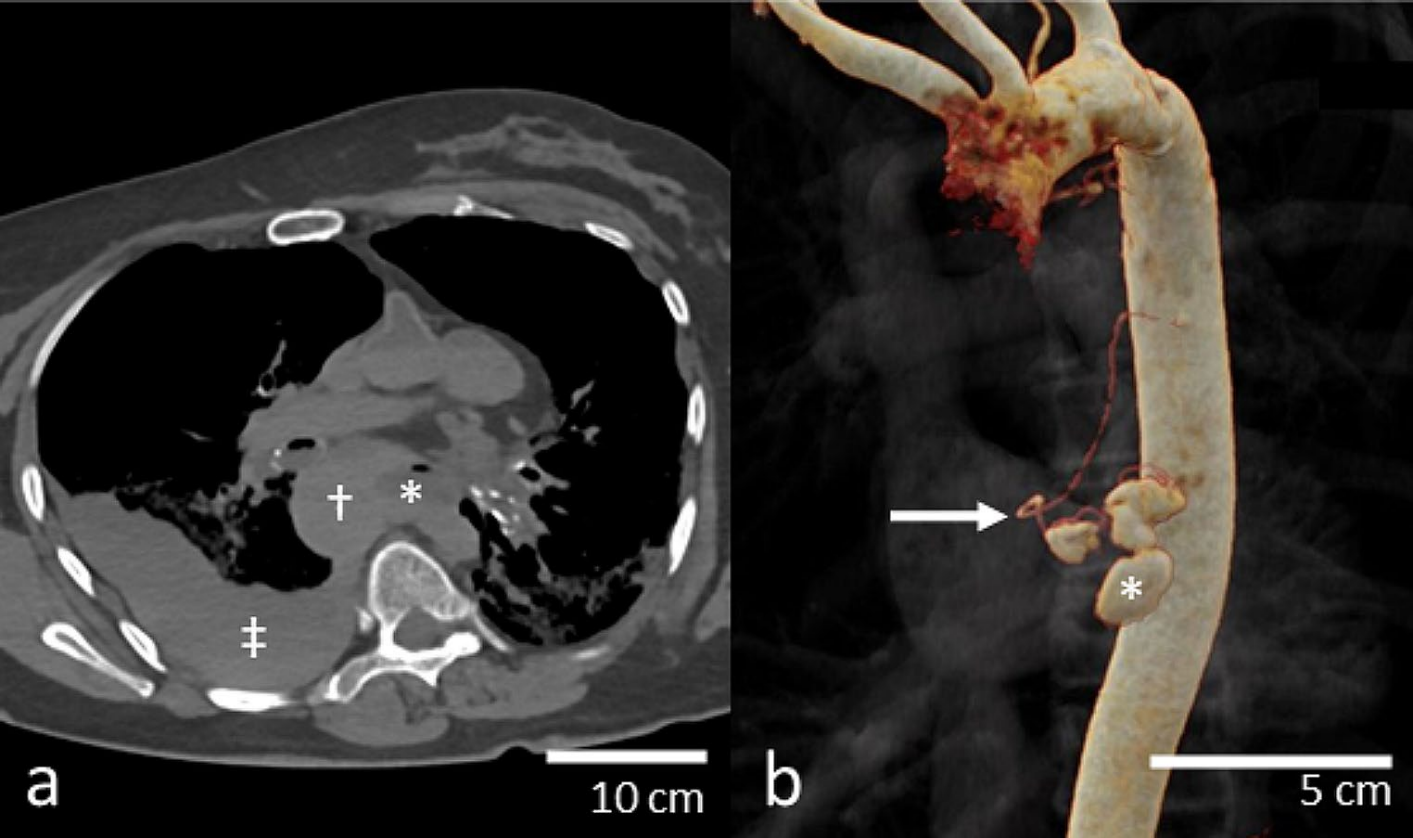
Post-mortem CT and post-mortem CT angiography findings. **(a)** Transverse reconstruction of the thoracic region of non-contrast post-mortem CT, demonstrating the esophagus (*) and paraesophageal hematoma (†), which is continuous with a substantial right-sided hemothorax (‡). **(b)** 3D-reconstruction of the arterial phase of the post-mortem CT angiography, demonstrating an esophageal branch of the descending aorta (arrow) with contrast leak into the mediastinal hematoma (*)

Relevant radiological findings included a large right-sided hemothorax and a longitudinal paraesophageal hematoma (Fig. 1a). These findings prompted further imaging via targeted post-mortem CT angiography of the mediastinal region.

PMCT angiography was undertaken using an in-house developed technique as previously described [1]. Both the femoral artery and vein on the side with the least calcific plaque, as identified on the PMCT, were exposed in the groin and cannulated with a 16 French (Fr.) pediatric arterial bypass cannula and 16 Fr. venous return catheter, respectively (Maquet Holding B.V & Co. KG, Rastatt, Germany). For arteriography, the femoral artery was connected to the connecting tube of a Dodge^®^ embalming pump with automatic pressure control (Model LPP-APC24, Dodge Co, MA, USA) and the venous catheter vented into a collecting bucket. The positioning was reversed for venography. Based on an average 70 kg patient, 150 mL of Omnipaque™ (Iohexol) 350 mg/mL (GE Healthcare, Little Chalfont, UK) iodine-based radiographic contrast was mixed with 1700 mL of a polyethylene glycol (PEG) 200 g/mol (Merck Group, Bayswater, Vic). Full body CT acquisitions were undertaken following each contrast solution infusion.

For the arteriogram, targeted imaging of the mediastinal region imaging showed adequate filling of the arterial circulation of the mediastinum. There was a contrast leak centrally into the mediastinal hematoma, originating from a small esophageal branch of the descending aorta (Fig. 1b and Electronic Supplement 1). The venogram showed adequate filling of the caval veins and right side of the heart, without further enhancement of the mediastinal hematoma. No other contrast extravasation was demonstrated.

## Autopsy findings

**Fig. 2:**
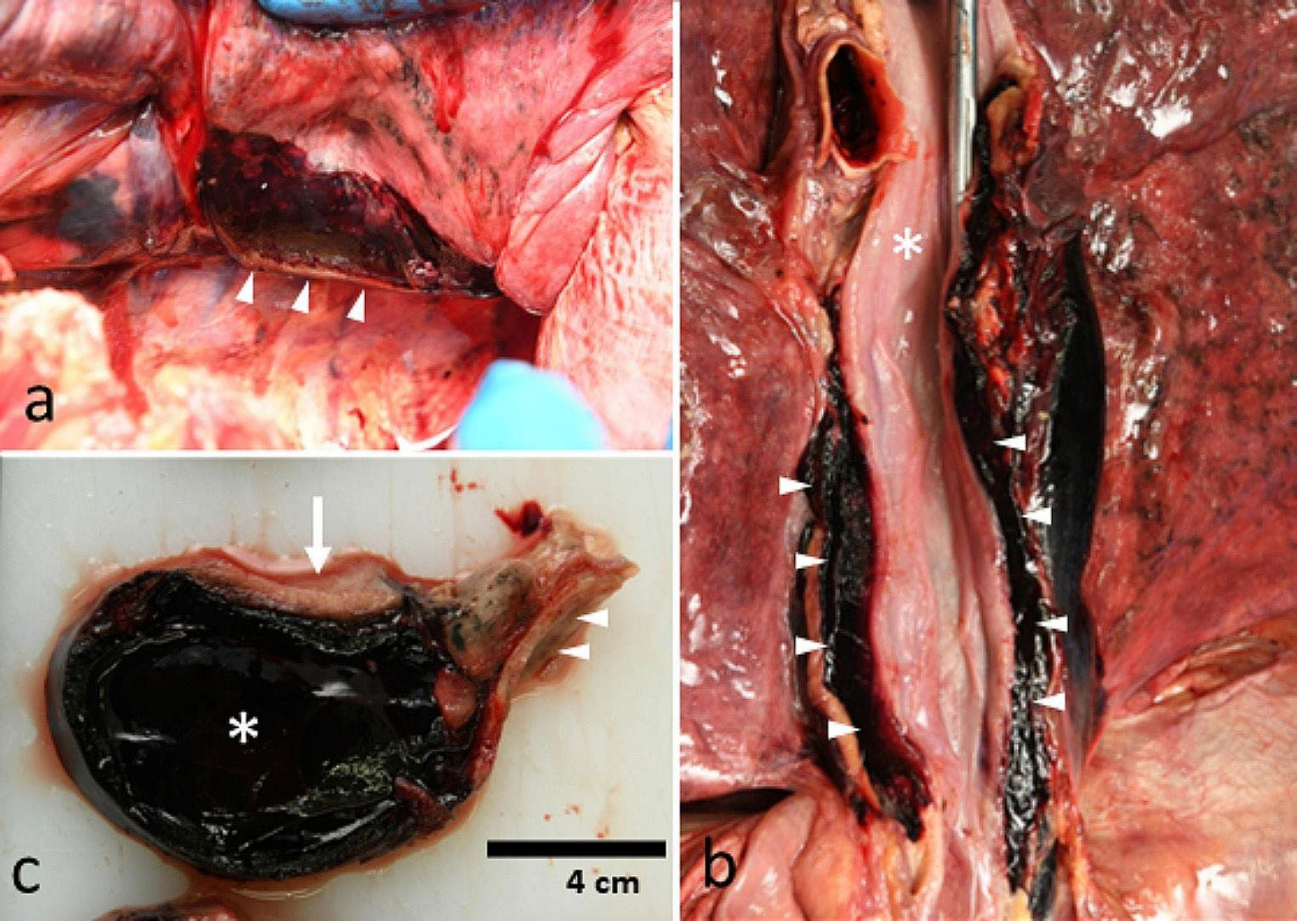
Autopsy findings. **(a)** The surface of the right side of the mediastinal pleura is exposed by lifting the right lung, demonstrating a rupture of the pleural (arrowheads) due to underlying hematoma. **(b)** Detail of the mediastinum after removal of the descending aorta and opening of the esophagus (*). The hematoma (arrowheads) is located longitudinally and directly adjacent to the esophagus. **(c)** Transverse sections of the hematoma after 48 h of formalin fixation. Note the homogenous aspect of the hematoma (*) and the close relation to the adjacent esophagus (arrow). In the right part of the image a part of the trachea is present (arrowheads)

Autopsy confirmed the presence of a large longitudinal paraesophageal hematoma. The hematoma was mostly contained by the mediastinal parietal pleura but had locally ruptured into the right pleural cavity (Fig. 2a). The right pleural cavity contained approx. 800 ml of blood and blood clots. The left pleural cavity contained approx. 150 ml of bloodstained watery fluid.

The thoracic and abdominal organs were removed *en bloc*. Surrounding the esophagus was an elongated hematoma of approximately 20 × 5 × 5 cm, directly adjacent to the esophageal muscular envelope (Fig. 2b). The esophageal mucosa was unremarkable. The aorta showed mild atherosclerosis but was otherwise unremarkable. All other mediastinal structures (e.g., caval veins, trachea, lymph nodes) were normal. Apart from emphysematous lungs, there were no other remarkable autopsy findings.

The mediastinal hematoma and adjacent tissues were placed in formalin for more detailed examination. After 48 h of formalin fixation, transverse sections of the hematoma revealed a large homogenous hematoma which was mostly directly peripheral to the esophageal muscular wall (Fig. 2c). There was no clear connection to other surrounding structures, such as the lungs, blood vessels or lymph nodes. There was no evidence of tumor of vascular malformation.

## Histological examination

Representative sections of the hematoma and adjacent structures were processed into routine hematoxylin and eosin-stained histological sections. Examination of these sections confirmed the presence of acute mediastinal hemorrhage without signs of organization. The hematoma was mostly situated outside the esophageal muscularis propria, with locally sparse hemorrhage in the submucosa.

## Toxicological analysis

Toxicological analysis of post-mortem blood detected a blood ethanol of 0.15 g/100 mL, and non-toxic/therapeutic levels of amisulpride (0.2 mg/L), paroxetine (0.1 mg/L) and paracetamol (5 mg/L). The effects of these substances were considered inconsequential for the cause of death.

## Discussion

The cause of death was believed to be directly related to the ruptured paraesophageal hematoma, with associated blood loss and impediment of lung function as main drivers within the mechanism of death. Pre-existing lung emphysema was deemed a contributing factor.

A large variety of underlying pathologies can precipitate mediastinal and/or paraesophageal hematoma. Traumatic causes include medical treatment such as endoscopy [2, 3], ingestion of foreign bodies [4] and various forms of blunt and penetrating trauma (e.g. [5, 6]). The case circumstances and post-mortem findings however excluded such an explanation. Mediastinal tumors or vascular malformations rarely cause large mediastinal hemorrhage [7–9] but were readily excluded by the post-mortem findings. The post-mortem examination furthermore excluded underlying aortic or bronchial pathology.

Given the medical history of gastro-esophageal reflux disease and close spatial relation to the esophagus, an underlying esophageal pathology was considered in more detail.

Esophageal varices usually rupture intraluminally and cause gastro-intestinal hemorrhage, but rare cases of externally ruptured varices have been described [10]. Still, autopsy and histology did not demonstrate esophageal varicose veins. In addition, no liver cirrhosis and no other, less common underlying causes of esophageal varices were noted (e.g., portal thrombosis or sarcoidosis).

Frequent or forceful vomiting can cause tears to the esophageal mucosa (Mallory-Weiss lesions) and these can present with esophageal hematomas, with or without total rupture of the esophageal wall [3]. The latter is also known as ‘Boerhaave syndrome’ named after the Dutch physician Herman Boerhaave, who described the entity in the 18th century. The post-mortem findings however demonstrated an intact esophageal lining.

Apoplexy (derived from the Greek *apoplēxía* meaning ‘to strike suddenly’) has been used since antiquity to medically describe the sudden onset of severe dysfunction, such as loss of consciousness or paralysis [11]. The term is historically associated with cerebrovascular accidents (cerebral apoplexia) but is also used to describe acute haemorrhage in other organs or anatomical locations with varying underlying pathologies. Current literature includes descriptions of abdominal, adrenal, pituitary, pulmonary, ovarian, and esophageal apoplexy [12–16].

Esophageal apoplexy is very rare form of esophageal hemorrhage and is also referred to as dissecting intramural hematoma of the esophagus (DIHE), dissecting hematoma, spontaneous intramural rupture, spontaneous intramural hematoma, and (idiopathic spontaneous) esophageal hematoma. The entity has been described in various clinical case reports and small case series (e.g., [2, 17–21]) and is characterized by the presence of an intramural and/or submucosal hematoma in the esophageal wall. Angiographic data suggests that the hematoma is supplied by esophageal branches of the descending thoracic aorta [22]. The cause is essentially unknown but is postulated to be due to sudden pressure changes in the esophagus and/or trauma. As such, its occurrence has been associated with vomiting, esophageal instrumentation, blunt chest trauma or foreign body ingestion. Many cases however remain idiopathic or seem to occur spontaneously. Bleeding diathesis or anticoagulation therapy is a common finding in such cases. Elderly women appear to be most often affected [17].

Based on the entirely of case circumstances and post-mortem findings, we believe our case is also best classified as esophageal apoplexy, although some important differences with the literature were noted.

The hematoma in esophageal apoplexy is usually described in the submucosa, between the muscularis mucosae and the muscularis propria. In our case, autopsy however demonstrated the hematoma to be largely situated outside the esophageal muscular envelope. Microscopic examination showed some sparse hemorrhage in the submucosa. It was considered that this may be residual blood from a larger submucosal hemorrhage which had ruptured/decompressed to outside the muscularis and into the thoracic cavity.

Esophageal apoplexy has almost invariably a benign course, with the symptoms and hematoma resolving after a few days to weeks of supportive care [17]. Severe complications are seldomly reported, and fatal cases and are even more rare:

Shim et al. reviewed 119 published cases, of which eleven developed severe bleeding complications and three eventually died [23]. These were all individuals of either advanced age or with severe comorbidities. In one case the death was not directly related to the esophageal apoplexy.

Folan et al. reviewed 32 cases of which two died [24]. One was an elderly individual which was also described by Shim et al. The other death was deemed to be unrelated to the esophageal pathology.

Beumer described a 78-year-old woman who died of a submucosal esophageal hematoma with esophageal perforation and extension of the hemorrhage into the left pleural space [17].

Isaac et al. described a case of a severely comorbid, elderly male with cardiovascular collapse due to gastro-intestinal hemorrhage secondary to submucosal esophageal hematoma [22]. The bleeding was treated by transcatheter embolization of an esophageal branch of the descending aorta, but the patient did not survive.

Pomara et al. reported an autopsy case of a 32-year-old woman with a laceration of the proximal esophageal wall and submucosal esophageal hematoma extending into the right pleural cavity [25].

Byard very briefly discussed esophageal apoplexy in a review on esophageal causes of sudden death [26]. He however did not refer to any fatal cases.

Fatal esophageal apoplexy thus appears to be very rare, and such fatal cases are generally associated with advanced age, severe comorbidities or a concomitant tear or perforation of the esophagus wall.

## Conclusion

We present a case of sudden unexpected death due to rupture of a paraesophageal hematoma which, after a comprehensive post-mortem examination, was deemed to be an atypical case of ‘esophageal apoplexy’. Our case highlights that although usually benign, esophageal apoplexy can present as sudden unexpected death.

## Electronic supplementary material

Below is the link to the electronic supplementary material.

Supplementary Material 1

## Data Availability

The data that support the findings of this study are available from the corresponding author upon reasonable request.
