# Supplementary figures and images for: An atypical case of fatal ‘esophageal apoplexy’: post-mortem findings and differential diagnosis
Source: Int J Legal Med. 2024 Jun 27;138(6):2351–5. doi: 10.1007/s00414-024-03280-6 (PMC11490419; doi:10.1007/s00414-024-03280-6)

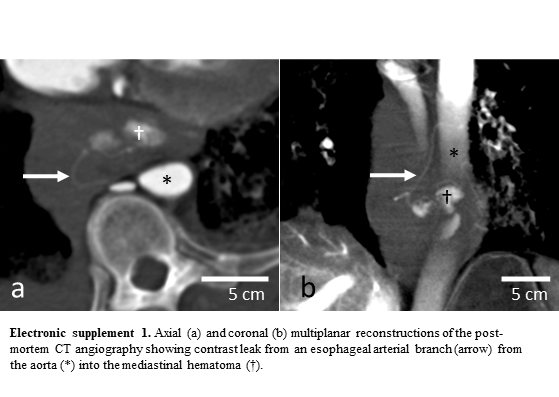

Supplement: Supplementary file 1 — Supplementary Material 1 [file 414_2024_3280_MOESM1_ESM.tif]
